# Adrenocorticotropic hormone gel in the treatment of systemic lupus erythematosus: A retrospective study of patients.

**DOI:** 10.12688/f1000research.7192.2

**Published:** 2016-02-24

**Authors:** Xiao Li, Josh Golubovsky, Joyce Hui-Yuen, Ummara Shah, Ewa Olech, Rosalia Lomeo, Vijay Singh, Howard Busch, Mary Jane Strandberg, Kayla Strandberg, Leslie Horowitz, Anca Askanase

**Affiliations:** 1 Division of Adult Rheumatology, Columbia University Medical Center, New York, NY, 10032, USA; 2 Division of Pediatric Rheumatology, Columbia University Medical Center, New York, NY, 10032, USA; 3 Division of Rheumatology, University of Rochester Medical Center, New York, NY, 14642, USA; 4 University of Nevada School of Medicine, Las Vegas, NV, 89102, USA; 5 Arthritis and Pain Center, Mary Washington Hospital, Las Vegas, NV, 89102, USA; 6 Arthritis Osteoporosis and Rheumatology Associates, Sewell, NJ, 08080, USA; 7 Family Arthritis Center, Jupiter, FL, 33458, USA

**Keywords:** ACTH, Acthar Gel, lupus treatment, SLE, steroid-sparing agent, Systemic Lupus Erythematosus

## Abstract

**Objectives**: Acthar Gel is a long-acting formulation of adrenocorticotropic hormone (ACTH) with anti-inflammatory effects thought to be mediated in part through melanocortin receptor activation. This study was initiated to understand the role of Acthar Gel in SLE treatment in rheumatology practices.

**Methods**: This is a retrospective case series of nine adult female patients treated with Acthar Gel for at least six months at five academic centers. Treating physicians completed a one-page questionnaire on lupus medications, disease activity, and outcomes. Clinical response was defined using SLEDAI 2K and improvement in the clinical manifestation(s) being treated.

**Results**: The most common clinical SLE manifestations/indications requiring therapy with Acthar Gel were arthritis, rash, and inability to taper corticosteroids. The mean SLEDAI 2K score at baseline was 5.8 ± 5.0 (range 0-16). Six patients were concomitantly treated with corticosteroids (mean dose 18.3mg/day). All patients were on background SLE medications including immunosuppressives. Seven of nine patients had an overall improvement, with a decrease in SLEDAI 2K from 5.8 ± 5.0 at baseline to 3.5 ± 2.7 (range 0-8); four of five patients had improvement or resolution in arthritis, and one of two patients had resolution of inflammatory rash. Four patients discontinued corticosteroids and one patient tapered below 50% of the initial dose by 3 months of treatment with Acthar Gel. No adverse events were reported.

**Conclusions**: This study suggests a role for Acthar Gel as an alternative to corticosteroids in the treatment of SLE. Acthar Gel appears to be safe and well-tolerated after 6 months of treatment, with a significant reduction in disease activity.

## Introduction

Systemic lupus erythematosus (SLE) is a chronic autoimmune disease of unknown etiology. The prevalence of SLE in the U.S. is estimated to be as high as 73 per 100,000 people ^1^. The hallmark of SLE is the production of autoantibodies directed against the patient’s own healthy tissue and organs. Clinically, SLE is characterized by flares, periods of increased disease activity and periods of quiescence ^2^.

Adrenocorticotropic hormone (HP Acthar® Gel, repository corticotropin injection, Questcor Pharmaceuticals, Inc., Union City, CA) is FDA-approved for the treatment of SLE ^3^. Acthar Gel was widely used in the 1950s as an effective therapy for SLE. However, it has been gradually replaced by synthetic glucocorticoid analogues. Acthar Gel is a long-acting formulation of adrenocorticotropic hormone (ACTH). ACTH is a 39 amino acid peptide that derives from post-translational processing of the precursor molecule proopiomelanocortin (POMC) and belongs to an anti-inflammatory group called melanocortins. Current understanding of Acthar Gel indicates that its effectiveness is a result of both its steroidogenic and direct anti-inflammatory effects through activation of different melanocortin receptors (MCRs) ^4– 6^. Acthar Gel binds to melanocortin receptors in a variety of cell types including immune cells. MC2R activation is responsible for the steroidogenic effects of Acthar Gel. Endogenous ACTH and Acthar Gel stimulate the adrenal cortex to secrete cortisol and other steroids by binding to the MC2R ^4^. The trophic effects of endogenous ACTH on the adrenal cortex are not well understood beyond the fact that they appear to be mediated by cyclic adenosine monophosphate (cAMP). The release of endogenous ACTH is under the influence of the nervous system via the regulatory hormone released from the hypothalamus and by a negative corticosteroid feedback mechanism. Elevated plasma cortisol suppresses pituitary ACTH release. Administration of exogenous ACTH via Acthar Gel can over-ride the negative feedback mechanism ^4^. There is a significant body of evidence as to which melanocortin receptor subtypes are involved in the direct anti-inflammatory effects ^7^. MCRs are expressed on virtually all the cells; the activations of MC1R, MC3R, and MC5R, in particular, are thought to be responsible for the direct anti-inflammatory effect. This is supported by experiments using MCR-selective synthetic analogs, and animal data ^4, 7– 11^. The MC1R-selective agonists inhibited tumor necrosis factor α (TNF-α)-induced activation of NF-κB and down-regulated expression and secretion of endothelial cell selectin, vascular cell adhesion molecule, and intercellular adhesion molecule in human dermal vascular endothelial cells treated with TNF-α ^4, 11^. In a study with adrenalectomized rats, Acthar Gel decreased experimental arthritis, indicating a steroid-independent action ^12^. 

A recently published open-label trial by Fiechtner and Montroy evaluated ten SLE female patients with persistent moderate-to-severe active disease while receiving standard therapy treated with Acthar Gel, 1 mL (80 IU/mL) by self-administered subcutaneous injection for 7–15 days. These patients showed significant improvement in the intensity of flares (primary endpoint), as measured by the SLE Disease Activity Index (SLEDAI 2K) score ^13^. Other clinical parameters also showed significant ( *p*<0.05) improvement, including physicians’ and patients’ global assessments, fatigue score measured by the Functional Assessment of Chronic Illness Therapy-Fatigue (FACIT-Fatigue) scale, and erythrocyte sedimentation rate ^13^.

SLE standard of care treatment uniformly includes corticosteroids. However, the potential for serious side effects from long-term corticosteroid use motivates the development of clinical trials with steroid-free regimens. The desired immune suppression of steroids can increase the risk of infections and metabolic effects such as diabetes, significant weight gain, Cushingoid habitus (moon face, buffalo hump, truncal obesity), striae, increased blood pressure, and fluid retention are concerning to both patients and doctors. Long-term steroid use can also lead to other serious consequences such as osteoporosis and fractures, avascular necrosis, and cataracts. Acthar Gel stimulates the production of endogenous steroids, the overproduction of which would have similar side effects as the synthetic steroids used in the current SOC regime. However, the combination of steroid-mediated immunosuppressive and direct anti-inflammatory effects of Acthar Gel provides a different approach in the management of SLE activity. This study was initiated to further understand the role of Acthar Gel in the treatment of SLE in several rheumatology practices.

## Methods

### Study design

This is a retrospective case series of patients from five academic/community clinical practices located in the United States. Five physicians from practices in Florida, Nevada, New York, and Virginia, agreed to participate in the study and complete a one-page questionnaire regarding the use of Acthar Gel in their patients with SLE. The study was approved by the Columbia University Medical Center Institutional Review Board and requisition of informed consent was waived.

### Patients

Patients in this study had been diagnosed with SLE by meeting at least four of eleven American College of Rheumatology (ACR) criteria or the Systemic Lupus International Collaborating Clinics (SLICC) classification criteria ^14, 15^. Based on the SLICC criteria, a patient is classified with SLE if four of the clinical and immunologic criteria are satisfied, including at least one clinical criterion and one immunologic criterion, or the patient has biopsy-proven nephritis compatible with SLE and with ANA or anti-dsDNA antibodies ^15^. All patients were between 18 and 88 years of age, had taken or were currently taking Acthar Gel to treat SLE and were followed by their physicians for at least 3–6 months since initiation of Acthar Gel treatment. All patients had been on stable doses of immunosuppressants, including five patients on oral corticosteroids, for at least four weeks prior to initiation of Acthar Gel therapy. All patients had failed to respond clinically to multiple immunosuppressants and/or were taking immunosuppressants at the time of Acthar Gel initiation. Patients who were not on stable doses of immunosuppressants, including corticosteroids, for at least four weeks prior to initiation of Acthar Gel were excluded. 

### Questionnaire

The questionnaire was a one-page survey that included information on demographics, SLE (treatment, disease activity, and laboratory results) and the administration of Acthar Gel (dose, duration of treatment, and side effects/adverse events). These data were recorded over six months of retrospective follow-up by the investigators, where the baseline questionnaire was recorded at time of initiation of Acthar by the treating physician. Physicians were asked to provide information on the clinical manifestations of patients being treated with Acthar Gel and whether those manifestations had improved at three and six months after initiation of therapy.

### Outcomes

Systemic Lupus Erythematosus Disease Activity Index 2000 (SLEDAI 2K) was calculated by the treating physician for each patient at baseline, 3 months, and 6 months after initiation of therapy with Acthar Gel. Improvement or resolution in the clinical manifestation(s) being treated was defined as follows: Improvement in arthritis was defined as a decrease by ≥50% in the number of swollen and tender joints and resolution was defined as the absence of swollen and tender joints. Improvement in rash was defined as a decrease in inflammatory rash by ≥50% of the affected area and resolution was defined as the absence of rash. These were in line with the SLEDAI 2K responder index (SRI-50), a modification of the SLEDAI that attempts to capture 50% improvement ^16^. *Per* Aspreva clinical trial guidelines, partial response for lupus nephritis was defined as ≥50% improvement in proteinuria and complete response as proteinuria lower than 500mg/24 hours ^17^. Improvement in all other initial symptoms driving the initiation of Acthar Gel was defined as ≥50% improvement per treating physician’s assessment. The definition of clinical response also included a decrease in ≥50% of the initial corticosteroid daily dose and/or discontinuation of corticosteroids during the time of treatment with Acthar Gel. Responders were the patients who achieved the outcome measures as defined above; non-responders were those who did not. Moreover, to be classified as a responder, there could be no worsening in other organ systems.

### Data analysis

Data were collected on demographic and disease characteristics, clinical manifestations requiring treatment, concomitant medications, disease course, and treatment outcomes. Due to small sample size, descriptive statistics were used where appropriate (Graphpad Prism 6, La Jolla, CA). Indications for the initiation of Acthar Gel treatment, mean values of dose, and frequency of Acthar Gel treatment were evaluated. Data on response to Acthar Gel treatment and adverse events were summarized.

## Results

Table 1 summarizes patient demographic data, disease history, and SLE clinical manifestations along with concomitant medications of the nine patients included in the study. Arthritis, inability to taper corticosteroids, and rash appeared to be the most common reasons for the initiation of Acthar Gel treatment. All nine patients had positive antinuclear antibodies (ANA) titers and the dose of 80 IU of Acthar Gel biweekly by subcutaneous injections was prescribed per recommended dosing ^18^. Acthar Gel was added in addition to the patients’ immunosuppressant and steroid regimens to better control disease activity and to allow for prednisone taper.

**Table 1.** Summary of patient demographic and systemic lupus erythematosus data.

Patient	Age (Years)	Disease Duration (Years)	Sex	Race/ Ethnicity	Concomitant Medications	Clinical Manifestation			SLEDAI 2K		
						Baseline	Month 3	Month 6	Baseline	Month 3	Month 6
1	30	11	F ^a^	Caucasian	Hydroxychloroquine Mycophenolate Mofetil Prednisone	Renal Inability to taper steroids	No improve Discontinue steroids	No improve Remained off	4	4	4
2	48	4	F	African- American	Hydroxychloroquine Mycophenolate Mofetil	Rash Arthritis Serositis CNS ^b^ Oral Ulcers	Resolution No improve Resolution Resolution Resolution	Resolution No improve Resolution Resolution Resolution	16	4	4
3	44	4	F	Hispanic	Methotrexate Belimumab Prednisone	Inability to taper steroids	Discontinue steroids	Remained off	0	0	0
4	49	16	F	Hispanic	Hydroxychloroquine Mycophenolate Mofetil	Renal	Resolution	Relapse	8	4	8
5	51	3	F	African- American	Methotrexate Prednisone Hydroxychloroquine	Arthritis Serositis Inability to taper steroids	Improved No improve Discontinue steroids	Improved No improve Remained off	10	6	6
6	31	8	F	Caucasian	Azathioprine Hydroxychloroquine Belimumab Prednisone	Rash Arthritis Inability to taper steroids	No improve Improved Discontinue steroids	No improve Resolution Remained off	6	6	2
7	48	19	F	Caucasian	Hydroxychloroquine Prednisone Belimumab Methotrexate	Arthritis Inability to taper Steroids	Resolution Tapered	Resolution Tapered	4	0	0
8	46	13	F	Asian	Belimumab Hydroxychloroquine	Arthritis	Improved	Improved	4	4	4
9	55	13	F	Hispanic	Azathioprine Hydroxychloroquine Belimumab Prednisone	Inability to taper steroids	Discontinue steroids	NA ^c^	0	0	NA

^a^F-Female, ^b^CNS-Central Nervous System, ^c^NA-Not Available.

Eight of nine patients had follow-up data available through six months of follow-up. Of these patients, five had arthritis, five were taking prednisone, two had rash, two had pleurisy, and two had active nephritis at baseline ( Table 1). The average corticosteroid dose at baseline was 18.3mg/day (range 7.5 to 40mg/day) and the average baseline SLEDAI 2K score was 5.8 (range 0 to 16). Patients with SLEDAI 2K scores of 0 were treated with Acthar Gel for inability to taper steroid, which was defined as requirement for prednisone treatment with >7.5mg for longer than 3 months despite repeated attempts at dose reduction. All other patients had persistent active disease.

There was an overall decrease by 40.2% in individual SLEDAI 2K scores in eight patients at Month 6. Two patients showed improvement in arthritis after six months of treatment, and two showed resolution of arthritis ( Table 1). One of the two patients with renal involvement at baseline achieved complete remission of lupus nephritis at 3 months but relapsed with return of proteinuria and active lupus nephritis at 6 months. Of the five patients who were initially on prednisone, four were able to completely stop prednisone at 3 months after the initiation of Acthar Gel and remained off corticosteroids at 6 months. The one patient who remained on prednisone was able to taper prednisone from 30mg/day to 6mg/day in three months and then 2mg/day after six months without worsening arthritis. One patient had only 3-month follow-up data available due to being lost to follow-up; for this patient, corticosteroids were discontinued at 3 months after initiation of Acthar Gel therapy.

Overall, seven of the nine patients in this study improved based on the ability to taper steroids, decrease in SLEDAI2K score, and the extent of their active clinical manifestations that had not resolved. Common side effects seen with use of Acthar Gel in other diseases include hypertension, hyperglycemia, increased susceptibility to infection, weight gain, and decreased bone density. However, no adverse effects were reported by any of the patients in our study while on treatment with Acthar Gel.

## Discussion

The current study evaluates the efficacy and safety of Acthar Gel in nine patients with SLE from five academic clinical practices. Treatment with Acthar Gel improved disease activity and allowed patients to taper and discontinue steroids without significant side effects. In recent years, there has been increased interest in treatment regimens that allow for lower corticosteroids doses in SLE due to concerns about the serious long-term consequences of chronic corticosteroid therapy which are responsible for some of the damage accumulation in SLE. While part of Acthar Gel’s efficacy is mediated through the production of steroids from the adrenal cortex, the non-steroidogenic anti-inflammatory effects via MCR activation are the most interesting. Studies in animal models of inflammation suggest responses to ACTH in the absence of adrenal steroid production ^12^. Additionally, Bomback *et al.* ^18^ have demonstrated previously that cortisol levels after administration of Acthar Gel were within normal limits, supporting a limited contribution for cortisol in responses to Acthar Gel, at least in this population of patients on long-term prednisone therapy. Plasma cortisol levels were not evaluated in our study.

A recently published open-label trial by Fiechtner and Montroy showed that Acthar Gel provided a significant reduction in SLE disease activity in ten patients ^13^. Despite the small sample sizes, patients showed improvement across all disease manifestations. Our data further substantiate the efficacy of Acthar Gel in patients with a variety of SLE manifestations and a role for ACTH as a steroid-sparing agent. Further studies in a larger population and for longer study duration will be of benefit to examine long-term outcomes and delineate adverse events.

Although data are limited, Acthar Gel appears to be well-tolerated, as no serious or unexpected adverse events were observed in our study patients, and the results of this study were consistent with historical observations and data from Fiechtner *et al.* ^13^. Fiechtner *et al.* ^13^ reported a sinus infection in one patient during the trial that resolved with antibiotic treatment, and another patient had bilateral edema in the lower extremities that resolved two weeks after the end of treatment.

We readily acknowledge several limitations of the study: the retrospective data collection, the small number of patients, the limited follow-up duration, and the lack of data on serum cortisol levels.

In conclusion, the results of this case series suggest that Acthar Gel is an effective therapeutic option for patients with SLE. Despite the limited number of patients, this study offers consistent results that afford an intriguing insight into the effectiveness of Acthar Gel in SLE. In particular, this study suggests that ACTH may be an attractive alternative to oral corticosteroids in the treatment of lupus; Acthar Gel may improve disease control through steroidogenic as well as direct anti-inflammatory effects, and does not appear to date to carry as severe a side effect profile as corticosteroids. Acthar Gel is currently being investigated in clinical trials enrolling patients with persistently active SLE, proliferative lupus nephritis (Class III, IV) administered together with mycophenolate mofetil, and in patients with membranous lupus nephritis (Class V). The results of these randomized, controlled trials are eagerly awaited ( NCT01753401, NCT02226341 and NCT01926054).
