# Supplementary material for: Adrenocorticotropic hormone gel in the treatment of systemic lupus erythematosus: A retrospective study of patients
Source: F1000Res. 2016 Feb 24;4:1103. Originally published 2015 Oct 23. [Version 2] doi: 10.12688/f1000research.7192.2 (PMC4850873; doi:10.12688/f1000research.7192.2)
Supplement: Peer review history

# Peer review: Referee response for version 2

**DOI:** 10.5256/f1000research.8799.r12633

**Published:** 2016-04-28

**Authors:** Anne Eberhard

**Affiliations:** 1 Department of Pediatrics, Division of Pediatric Rheumatology, Steven and Alexandra Cohen Children's Medical Center of New York, North Shore-LIJ Health System, New York, NY, USA

Comments noted

I have read this submission. I believe that I have an appropriate level of expertise to confirm that it is of an acceptable scientific standard.

# Peer review: Referee response for version 1

**DOI:** 10.5256/f1000research.7748.r11900

**Published:** 2016-02-19

**Authors:** Anne Eberhard

**Affiliations:** 1 Department of Pediatrics, Division of Pediatric Rheumatology, Steven and Alexandra Cohen Children's Medical Center of New York, North Shore-LIJ Health System, New York, NY, USA

The authors describe 9 patients who were treated with an adrenocorticotropic hormone - Acthar Gel for their active SLE.

This report is a retrospective study from 5 academic centers describing the effectiveness of the gel in 9 patients with SLE. This begs the question how was the study initiated?

The article should really be limited to 8 patients as Patient 9 was lost to follow up and therefore did not satisfy the described inclusion criteria.

The authors list the limitations of their study in the discussion.

The following are questions that arise from the paper

1. What and how many patients were excluded from the study as not meeting study criteria. In other words the patients included all seemed to do well on the Acthar Gel, however were there patients who were on the gel for less than 6 months (thereby not satisfying inclusion criteria ) who did not do well.
2. What were the actual doses of the gel - a mean dose is listed without any standard deviation.
3. As this is a retrospective study the listing of improvement, especially the definition of 50% improvement would have to be a guess as this would not necessarily be recorded in the patient's chart.
4. While it is a small sample size the statistics should include a mean and standard deviation. This is especially the case when the authors list the improvement of the SLEDAI 2K decreasing 3.5, what is this decrease from? There is one significant outlier a sick patient with an initial SLEDAI of 16 who after using the Acthar Gel reduced the SLEDAI to 4, although she was the only one whose arthritis did not improve on Acthar Gel administration.
5. What is the real benefit from using this medication. After all one is merely changing from one type of steroid to another. Is the benefit the ease of administration, less side effects or less long term complications

It will be of interest to see the results of the current clinical trials in SLE nephritis, in the 2 patients in this study with nephritis the Acthar Gel was either ineffective or its effects wore off .

I have read this submission. I believe that I have an appropriate level of expertise to confirm that it is of an acceptable scientific standard, however I have significant reservations, as outlined above.

# Response

**Published:** 2016-02-21

**Authors:** Joyce Hui-Yuen

**Affiliations:** Columbia University Medical Center, USA

Thank you very much for your review. We have submitted a new version and include the following in response to your comments:

*Comment*: What and how many patients were excluded from the study as not meeting study criteria. In other words the patients included all seemed to do well on the Acthar Gel, however were there patients who were on the gel for less than 6 months (thereby not satisfying inclusion criteria ) who did not do well.

*Response*: We have modified the timecourse to 3-6 months of Acthar Gel treatment to include all 9 patients, and apologize for any confusion.

*Comment:* What were the actual doses of the gel - a mean dose is listed without any standard deviation.

*Response*: We have removed the word 'mean' from the dosing as all patients were on 80 IU subcutaneously biweekly as per prescribing recommendations.

*Comment:* As this is a retrospective study the listing of improvement, especially the definition of 50% improvement would have to be a guess as this would not necessarily be recorded in the patient's chart.

*Response*: The improvement in outcomes was based on the SRI-50, which attempts to capture a 50% improvement in the patient's condition. This was recorded by the treating physician at the treatment site, and did not involve the central site (i.e., Columbia University) which received all surveys for analysis.

*Comment*: While it is a small sample size the statistics should include a mean and standard deviation. This is especially the case when the authors list the improvement of the SLEDAI 2K decreasing 3.5, what is this decrease from? There is one significant outlier a sick patient with an initial SLEDAI of 16 who after using the Acthar Gel reduced the SLEDAI to 4, although she was the only one whose arthritis did not improve on Acthar Gel administration.

*Response*: In the Abstract, we have modified the baseline and 6-month SLEDAI reports to include the standard deviations and ranges. So the decrease to a mean of 3.5 +/- 2.7 was from the mean baseline of 5.8 +/- 5.0.

*Comment:* What is the real benefit from using this medication. After all one is merely changing from one type of steroid to another. Is the benefit the ease of administration, less side effects or less long term complications.

*Response*: This is an excellent question - we also eagerly await the results of the clinical trials in active SLE, and lupus nephritis patients. At the present time, we have included the following sentence in the last paragraph of the Discussion in an attempt to clarify our conclusion: "In particular, this study suggests that ACTH may be an attractive alternative to oral corticosteroids in the treatment of lupus; Acthar Gel may improve disease control through steroidogenic as well as direct anti-inflammatory effects, and does not appear to date to carry as severe a side effect profile as corticosteroids."

Thank you again for your time and critical comments. We very much appreciate your review.

# Peer review: Referee response for version 1

**DOI:** 10.5256/f1000research.7748.r10908

**Published:** 2016-02-01

**Authors:** Giovanni Franchin

**Affiliations:** 1 Center for Autoimmune and Musculoskeletal Disease, Feinstein Institute for Medical Research, Bronx - Lebanon Hospital Center, Manhasset, NY, USA

Xiao Li *et al *present a well written report on a retrospective series of nine patient with active lupus and the effect of ACTH gel. Although the study is limited by the small number of subjects they include a diverse patient population the majority of which had very active disease with an average baseline SLE Disease Activity Index 2K of 6 and on combination immunosuppressive therapy. Moreover, the patients represent our usually more challenging patients with inability to taper steroids and multiple organ involvement. The authors were careful to select patients who had been initiated in ACTH gel and had not had any recent adjustment of immunosuppressive therapy, which is a common confounder of disease response/activity. The results in terms of tapering down/off steroids is compatible with our understanding of the steroidogenic effect of Acthar gel. The improvement of SLEDAI 2K was seen for half of patients. It would have been reassuring if data on individual dosing of Acthar gel had been given for all patients and confirmation of drug administration

.

Overall this study offers strong observational data to support further studies into the effectiveness of ACTH analogues in the treatment of lupus.

I have read this submission. I believe that I have an appropriate level of expertise to confirm that it is of an acceptable scientific standard.

# Response

**Published:** 2016-02-04

**Authors:** Joyce Hui-Yuen

**Affiliations:** Columbia University Medical Center, USA

Thank you very much for your review. We appreciate your time in reviewing our article and your critical comments. We apologize for any confusion regarding the dose of Acthar, which was, in fact, 80 IU subcutaneously twice weekly in all patients. Unfortunately, administration of the drug was not confirmed in this study.

Thank you again - we very much appreciate your time and effort in reviewing our article.
